# Bayesian logical neural networks for human-centered applications in medicine

**DOI:** 10.3389/fbinf.2023.1082941

**Published:** 2023-02-15

**Authors:** Juan G. Diaz Ochoa, Lukas Maier, Orsolya Csiszar

**Affiliations:** ^1^ Data Science & Machine Learning Division, PERMEDIQ GmbH, Wang, Germany; ^2^ Faculty of Electrical Engineering and Computer Science, Hochschule Aalen, Aalen, Germany; ^3^ John von Neumann Faculty of Informatics, Óbuda University, Budapest, Hungary

**Keywords:** explainable AI, electronic health records, epistemic uncertainty, logical neural networks, bayes neural networks

## Abstract

**Background:** Medicine is characterized by its inherent uncertainty, i.e., the difficulty of identifying and obtaining exact outcomes from available data. Electronic Health Records aim to improve the exactitude of health management, for instance using automatic data recording techniques or the integration of structured as well as unstructured data. However, this data is far from perfect and is usually noisy, implying that epistemic uncertainty is almost always present in all biomedical research fields. This impairs the correct use and interpretation of the data not only by health professionals but also in modeling techniques and AI models incorporated in professional recommender systems.

**Method:** In this work, we report a novel modeling methodology combining *structural explainable* models, defined on Logic Neural Networks which replace conventional deep-learning methods with logical gates embedded in neural networks, and Bayesian Networks to model data uncertainties. This means, we do not account for the variability of the input data, but we train single models according to the data and deliver different Logic-Operator neural network models that could adapt to the input data, for instance, medical procedures (Therapy Keys depending on the inherent uncertainty of the observed data.

**Result:** Thus, our model does not only aim to assist physicians in their decisions by providing accurate recommendations; it is above all a user-centered solution that informs the physician when a given recommendation, in this case, a therapy, is uncertain and must be carefully evaluated. As a result, the physician must be a professional who does not solely rely on automatic recommendations. This novel methodology was tested on a database for patients with heart insufficiency and can be the basis for future applications of recommender systems in medicine.

## 1 Introduction

In daily business, physicians are confronted with the constant integration and evaluation of different parameters to assess the patient’s condition and in this way, establish correct diagnoses as well as therapies, which are standardized and encoded as Therapy Keys (TKs). This assessment is particularly difficult for health professionals due to the constant work overload in health centers. In addition, there is a cognitive impairment that represents selecting an item, like a potential therapy, from a portfolio with several options (Hall and Walton, 2004). To this end, support systems represent the most effective option to assist the daily work of health professionals; but these systems require mathematical models.

Traditionally, such support systems have been defined as expert systems (Sandell and Bourne, 1985), (Zhou et al., 2021). But recently, with the development of efficient computing methods, the use of deep learning methods has found more acceptance, for instance for pattern recognition not only of medical images but also of data contained in EHRs for the development of, for instance, recommender systems in medicine (Rajkomar et al., 2018). Any model used in medicine must, however, be explainable, i.e., the customer must be able to understand how the results can be obtained.

Conventional methods of machine learning, like decision trees, are essentially explainable since the derivation of the final result can be tracked throughout the entire computation process. However, these methods are limited by their accuracy and scalability, i.e., their ability to handle an ever-growing amount of information. Deep learning methods are an attractive option over other modeling alternatives. The methods based on this method are unfortunately unexplainable, since the computation of the network’s weights follows internal coupled optimization processes that are difficult to explain and present to customers with little technical background in the field. To this end, significant effort has been made in order to establish and standardize explainability in deep learning (Linardatos et al., 2020a).

Alternatively, deep-learning models can be explainable with a change in the network’s structure, for instance by combining neural networks with continuous logic and multi-criteria decision-making tools (Csiszár et al., 2020) leading to the definition of Logical Neuronal Networks (LONNs). Recently, this methodology has been applied to recommender systems in medicine, providing the option to define the logical combination of a hierarchy of parameters (Ochoa et al., 2021). However, one limitation of this methodology is its inherent inflexibility, which could be responsible for the low performance of this modeling methodology (Wang, 2021). Medical customers desire control over the logical combinations, and the smaller and better-defined as well as more generalizable the network is, the easier it is to make it understandable to them, perhaps at the expense of precision.

An explainable model must also manage uncertainties in the data. The recording of health data is prone to uncertainties and errors, coming from the inherent biological variability (considering that every organism is exposed to an environment, implying the recording of data in non-controlled experimental conditions), to errors in the recording of diagnoses (which is about 63% in EHRs^1^), leading to a persistent epistemic uncertainty in the recorded data in EHRs, which is present in all biomedical research (Viale, 2021). This implies that models should be essentially non-deterministic and that the model must be stochastic, rather than flexible or “smart” in its architecture. Thus, an explainable model must be able to tell the customer how high its precision is by indicating the corresponding amount of uncertainty^2^. Explainable models are not only concerned with transparency of the structure and information flow, leading to a final prediction based on simple, generalizable models (Occam’s razor), but also imply the possibility of generating parallel models reflecting this uncertainty.

Now we combined LONNs with Bayesian Neural Networks (BNNs) (Lampinen and Vehtari, 2001) to create a new modeling methodology known as BaLONNs. To represent uncertainty and imprecision in real data, previous efforts have combined fuzzy logic with Bayesian Networks (Pan and Bester, 2018), but no implementation has been made in the context of deep learning.

We tested this novel approach for the prediction of the kind of heart failure (HF) and the expected therapy time (TL) of patients with diabetes, using a Pakistan Data Base from the UCI repository (Chicco and Jurman, 2020). Based on these results, we now aimed to develop not only an improved explainable model but also a human-centered application that informs the customer when the model is “unsure” about a given prediction.

This article is organized as follows. In the next section, we introduce the methodology and modeling strategy. Thereafter, we report testing the methodology and provide qualitative as well as validation results. Finally, we discuss the implications of the introduced methodology and provide an outlook on the next research steps.

## 2 Methodology

### 2.1 Data extraction, feature engineering and data balancing methods

This analysis uses data from diabetic patients, some of whom have heart failure (Pakistan Database, from the UCI repository^3^). The corresponding attributes are listed in Table 1, where V1 to V10 are the inputs, and O1, as well as O2, are the model targets. Observe that the parameter “Time” is a metric of the total time (in months) that the patient has been treated. In the recorded data we are not dealing with the variability of several measurements of a single parameter for one patient (aleatoric uncertainty), or the complete uncertainty about the internal processes of the system (ontological uncertainty), but with the implicit variations between patients contained in the data (epistemic uncertainty), which have different origins: from biological/physiological variability to systematic errors in the recording of the data.

**TABLE 1:** Principal input and output parameters extracted from the HER of diabetic patients with heart insufficiency.

Input/Output	Variable	Abbreviation	Kind of parameter
-	ID		Character
V1	Sex		Binary
V2	Age		Real value
V3	Creatinine_phosphokinase	CPh	Real value
V4	Ejection_fraction	EF	Real value
V5	High blood pressure	HBP	Binary
V6	Platelets	P	Real value
V7	Serum_creatinine	SC	Real value
V8	Serum_sodium	SS	Real value
V9	Smoking		Binary
V10	Anemia		Binary
O1	Diagnose (kind of heart insufficiency)	HF	Binary
O2	Time (feedback period)	TL	Multiclass

Since we required a large population, we synthesized additional patients from the original database. Personal information, like identification number (ID), age, and sex, was also correspondingly modeled. For the simulation of the distribution of diagnoses, we used the “synthpop” package^4^, basically using linear regression models for each parameter (Nowok et al., 2016).

From this data we can extract two main features.

• the kind of heart failure *HF* as a binary value representing patients with systolic heart failure (SHF, *HF = 1)* and heart failure with preserved ejection fraction (HFNEF, *HF = 0)*
• the therapy length *TL*, representing the time the patients are treated. In this last case, we transform the registered time τ , which is a natural number τ ϵ N (number of months), into a discrete scale representing low (*TL = 0*, for 0–1 month), medium (*TL = {1,2},* for 2–4 months), and a large (*TL = 3* for 5 and more months), expected therapy time.

While the first parameter *HF* is related to the kind of therapy a patient becomes, the second one, *TL*, is related to the quality of this therapy, such that a low-quality therapy corresponds to *TL = 0*, a medium-quality therapy corresponds to *TL = 1* or *TL = 2*, and a high-quality therapy corresponds to *TL = 3*. Therefore, this one is a problem with two different tasks (TL, HF) and can be defined as a Multitask Learning (MLP) problem, where multiple tasks are simultaneously learned by a shared model.

To solve this problem, we opted to implement the model as a regression algorithm. *This seems a natural strategy because regression algorithms, by definition, have a notion of the relative distance of target values* (Crawshaw, 2020).

**FIGURE 1:**
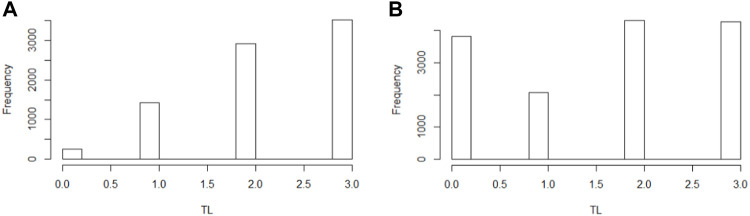
Distribution therapy length in the population in the original population **(A)** and the SMOTE balanced population **(B)**.

Since these classes are essentially imbalanced, we require balancing methods to reduce bias in our modeling. To this end, we implemented Synthetic Minority Oversampling TEchnique (SMOTE) to both *HF* and *TL* to create an oversampling of less frequent values. Despite its limitations, we selected this method because, according to Blagus et al., it is beneficial for low-dimensional data (Blagus and Lusa, 2013). In Figure 1, we present the original data distribution of *TL* before and after data balancing with SMOTE.

80% of the data is used exclusively for model training and validation. The final 20% of the data is test data used to evaluate the quality of the model. For both model training and quality evaluation, we normalized the input data.

### 2.2 Baseline models

Our baseline model is essentially a deep-learning model with dense fully interconnected neuron layers implemented on TensorFlow.

The basic architecture of the baseline model is resumed in Table 2, which has been selected to compare its performance against the LONN architecture. Observe that in this model we have implemented rectified linear layers, using MSE (Mean Square Error) as a loss function. Recent investigations have demonstrated that rectified linear functions are the most effective in representing data processing in neural networks^5^, particularly for networks with many layers (Urenda, 2020). Additionally, the ordinal classification problem of the therapy length is, based on Kramer et al., handled as a regression problem with an additional post-processing step (Kramer et al., 2001).

**TABLE 2:** Model parameters of baseline model.

Layer	# Units	Activation function
Input Layer	10	Relu
#1	10	Relu
#2	4	Relu
#3	3	Relu
#4	2	Sigmoid

### 2.3 Bayesian Neural Networks (BNN)

Often, errors occur in the way data is entered into EHRs. This is in part because the information in EHRs is partially manually curated, implying epistemic uncertainty in the stored data^6^.

To correctly model this uncertainty, we implement models that *marginalize the distribution of parameters in order to make prediction* ^7^ by implementing the weights in the neuronal network as a distribution defined as a Gaussian process. In this way, a trained model is not the result of the optimization of single parameters, but the optimization of the statistical distribution of these parameters. The same network with finitely many weights is known as a Bayesian neural network^8^. To this end, we aim to minimize the evidence lower bound of the network weights, which is defined as^9^ ^,^ ^10^

$$L\left(w_{lk}\right)=H\left(Q\left(w_{lk}^{\prime }\right)\right)-H\left(Q\left(w_{lk}\right);P\left(w_{lk},w_{lk}^{\prime }\right)\right),$$ (1)

where $H\left(Q\left(w_{lk}^{\prime }\right)\right)$ is the cross entropy defined as $H\left(Q\left(w_{lk}^{\prime }\right)\right)=-\underset{Z}{\sum }Q\left(w_{lk}^{\prime }\right)∙\log\left(Q\left(w_{lk}^{\prime }\right)\right)$ , Q is a distribution over unobserved variables $w_{lk}^{\prime }$ , in this case, the prior, and $P\left(w_{lk},w_{lk}^{\prime }\right)$ is the posterior of the distribution of observed data $w_{lk}$ , defined as a likelihood function. In this specific implementation, the observed data $w_{lk}$ are the neural network’s weights of the layer l to the layer k , and $w_{lk}$ is the estimated distribution for these weights. This definition is equivalent to minimizing the Kullback-Leiber divergence $D_{KL}\left(Q∥P\right)$ of the distributions Q and P .

*Thus, the network will be trained such that* $D_{KL}\left(Q∥P\right)\to 0$ *, as well as maximize the probability of the data under the posterior weights* ^11^ *:* the model fit the actual achieve high log-likelihood, while it stays close to the prior^12^. We define both $Q\left(w_{lk}^{\prime }\right)\left[\sigma_{\mathrm{pr}}\right]$ and $P\left(w_{lk},w_{lk}^{\prime }\right)\left[\sigma_{po}\right]$ as Gaussian distributions, where $\sigma_{\mathrm{pr}}$ is the corresponding standard deviation of the prior, and $\sigma_{po}$ is the standard deviation of the posterior. Finally, in all the probabilistic models, the last layer delivers the result as a distribution, with its standard deviation; in all the experiments we have fixed this standard deviation to 1.0. The implemented loss function is the log-likelihood multiplied by negative one *(negloglik*), which returns the value of the negative loglikelihood function for the data used to fit the probability distribution^13^ (the learning rate is listed in Table 3). The optimization process for the model training was computed using the Adam method (Kingma and Ba, 2017).

**TABLE 3:** Examples of logical operators and their corresponding implementation.

Logical operation	$w_{ij}$	$b_{i}$
AND	1	−1
OR	1	0
NOT (x)	−1	1
NOT (y)	−1	1
Not (x) and Not (x)	−1	1

The implementation has been performed on TensorFlow in R using R-Studio. The final trained model is an object containing the training functions for the distributions in the statistical layers. These Bayes layers are then used to evaluate the effect of epistemic uncertainty in two models.

• BNN: Bayesian Neural Networks (stochastic baseline model).
• BaLONN: Bayesian LONN.

### 2.4 LONNs and bayesian layers

**FIGURE 2:**
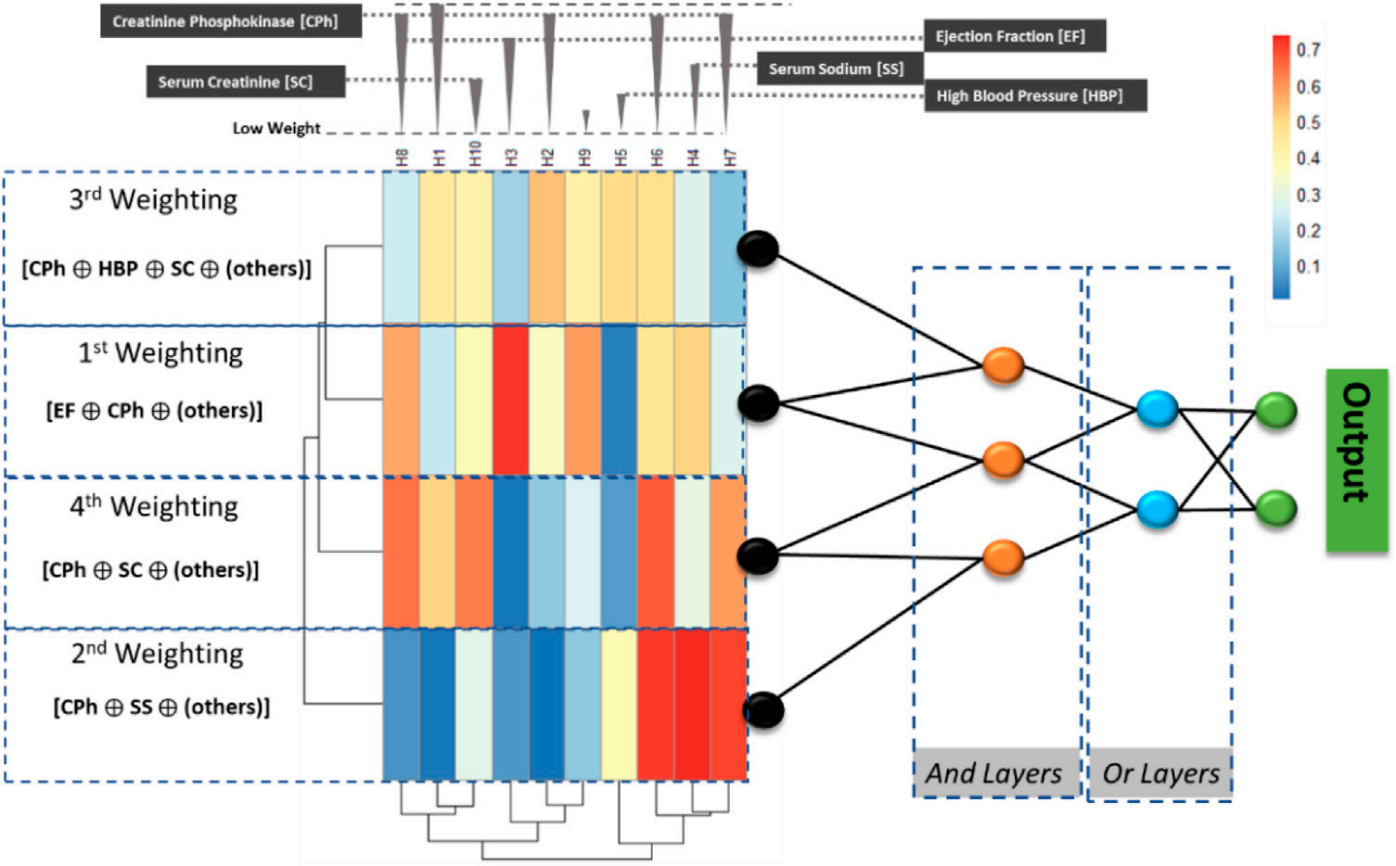
The implemented ProbLONNs contain distributed weights in the initial layer representing the weight distribution. The number of neurons in the hidden layers is defined according to the parameters presented in Table 2. In a multiclass problem, the implemented model tries to predict between, for instance, two classes based on the logical decision after combining parameters that have been identified by relevance depending on the weight estimated in the first model layers.

The application of LONNs for the evaluation of medical data has been described by Ochoa et al. (Ochoa et al., 2021), *whereby single layers are replaced by frozen weights and bias representing logical operations.* Here, a single Perceptron in the NN network is activated by so-called Squashing activation functions, a differentiable and parametric family of functions that satisfy natural invariance requirements and contain rectified linear units as a particular case (Urenda, 2020). These activation functions are in this framework defined as follows:

$$S_{\beta }\left(x\right)=\frac{1}{\beta }\ln\left(\frac{1+e^{\beta ∙x}}{1+e^{\beta ∙\left(x-1\right)}}\right),$$ (2)

where β is a real non-zero value that must be adjusted to let the model be convergent. Thus, the Perceptron in the neural networks’ hidden layers can model a threshold-based nilpotent operator (Csiszár et al., 2020): a conjunction, a disjunction, or even an aggregative operator. *This means that the first (and last layer) are non-frozen, i.e., only these two layers will be trained*, while the hidden layers of the pre-designed neural block, work as logical operators with frozen weights and biases. An intuitive idea of the combination of the Bayes Layers on the LONNs is presented in Figure 2, where the first two layers perform a weighting of the input parameters.

To visualize the model explainability, we exemplarily show the way how the LONN model performs a computation with the parameters listed in Table 1: Observe that the Creatine_Phosphokinase (CPh) has a high statistical weight in the model; thus, this parameter, combined with other parameters having also a high weight in the first layers, are combined in the logical gates to predict the two outcomes, HF and TL.

Thus, the baseline model is modified by freezing the weights and biases in the layers that are modeled as logical switches (see the parameters listed in Table 2). This definition implies that the number of trainable parameters for weight estimation gets reduced, also implying a reduction of the degrees of freedom of the model.

According to this, the following exemplary logical combination is performed using a LONN to predict Heart Failure (HF) and Therapy Length (TL) (abbreviations according to Figure 2; Table 1) [Creatin Phosphokinase, High blood pressure, Serum Creatinine, others] AND [Creatinine Phosphokinase, Ejection Fraction, others] OR [Creatin Phosphokinase, Serum Creatinine, others] AND [Creatinine Phosphokinase AND Ejection Fraction], where “others” refer to other parameters with a lower weight. Using logical notation, the logical combination of parameters can be described in the following way:

$$\left(\left[CPh⊕HBP⊕SC⊕\left(others\right)\right]\wedge \left[EF⊕CPh⊕\left(others\right)\right]\right)\vee \left[CPh⊕SC⊕\left(others\right)\right]\wedge \left[EF⊕CPh⊕\left(others\right)\right]$$ (F-1)

$$\left(\left[CPh⊕SC⊕\left(others\right)\right]\wedge \left[EF⊕CPh⊕\left(others\right)\right]\right)\vee \left(\left[CPh⊕SS⊕\left(others\right)\right]\wedge \left[CPh⊕SC⊕\left(others\right)\right]\right)$$ (F-2)

Observe that all the above-documented logic operators are fuzzy, i.e., logical operations are not exact but essentially fuzzy due to the implemented continuous-valued operators. In addition, because the logical layers are essentially frozen, there is little chance that the model will be overfitted if multiple layers are coupled into the model.

**FIGURE 3:**
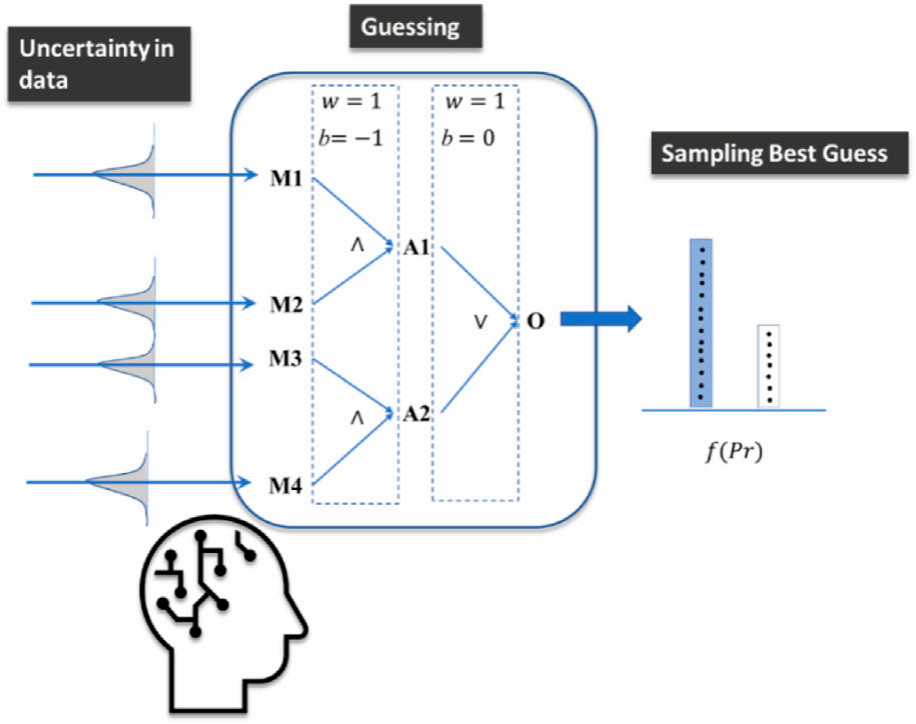
The implemented model mimics cognitive processes under uncertainty, where guesses are performed and finally sampled considering different options (outputs) to take a final decision. While a decision is made, other alternative answers are not fully discarded.

Our implemented Logic-Operator neural network (LONN) is thus a method simulating the cognitive logical thinking process (Figure 3).

We simulate human thinking by introducing probabilistic layers (Sohn and Narain, 2021) which recognize not only Bayesian inference but also their inherent uncertainty, as well as the possibility of representing both uncertainty and inaccuracy (Pan and Bester, 2018). Therefore, the results of the model are not intended to present accurate solutions and results. Rather, these results represent both a plausible prediction of an outcome and its corresponding uncertainty.

**FIGURE 4:**
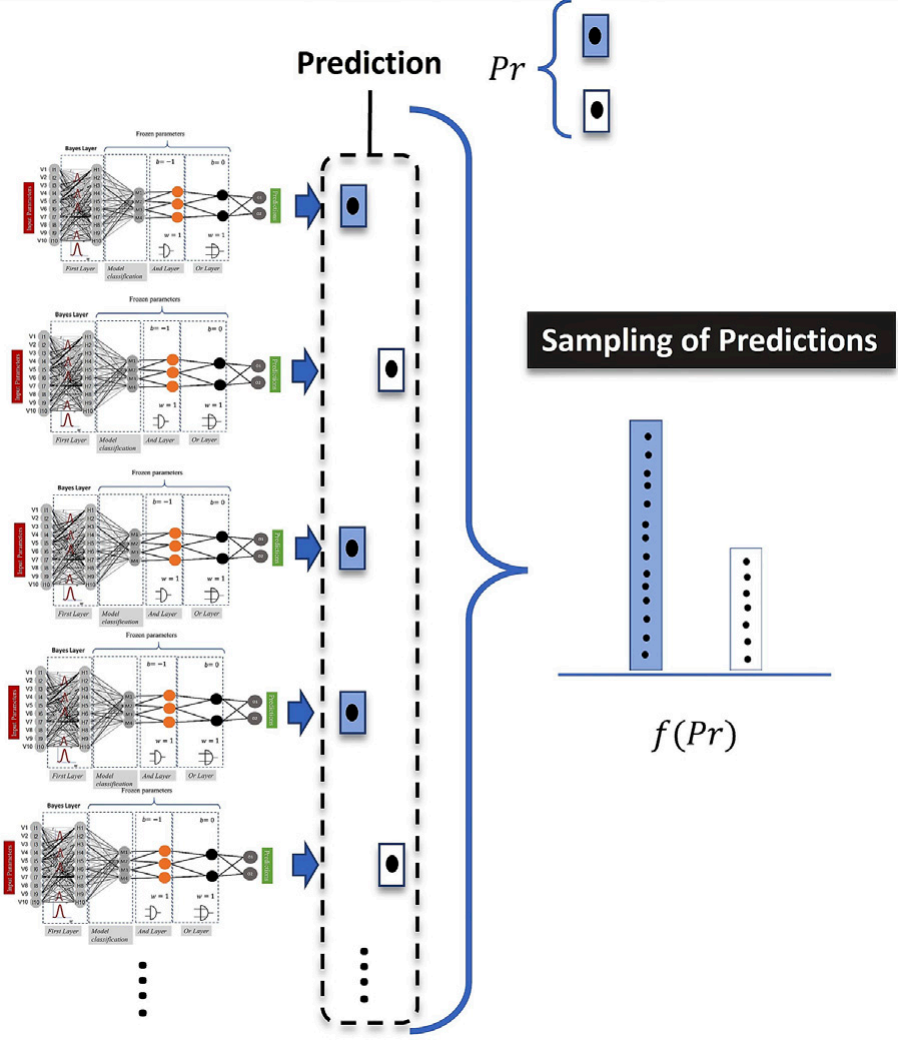
Intuitive meaning of the application of Bayes in the model definition. For the computation of a single prediction, the computed results are sampled.

By plotting this cognitive process, we are dealing not only with a single model prediction but with the sample of results provided by models with different weights provided by the Bayesian model (see Figure 4). Accordingly, the mathematical implication of combining LONNS with Bayesian networks is that the possible fuzzy logic combinations are not unique but distributed. In terms of the explainability of the model, this means that the fuzzy logic combinations provided (e.g., Figure 1 and Figure 2) are the mean of all averaged possible combinations identified by the model.

## 3 Results and model validation with variability

For the model definition we performed a systematic evaluation of the following parameters.

• Normalization, for instance treatment times and other parameters.
• Meta parameter analysis.
o Dependency of the model on activation functions.
⁃ Dependency of model distributions on activations.
⁃ Dependence of model distributions on LONNs.
o Learning Rate—Adam.
o Number of neurons.
o Number of epochs.
• Architecture analysis—especially for LONNs.
• Statistics—distribution functions in the Bayesian strata—possibly new definition of the model.

Furthermore, we performed a systematic analysis of the influence of the parameters in the Bayesian layers on the model precision (standard deviation in the prior and posterior standard deviations). Based on the previous definitions, we performed different computations using the meta-parameters for each model listed in Table 4 considering Deep Neural networks (DNN) and Bayes Neural Networks (BNN).

**TABLE 4:** Main model parameters used in the current implementation.

	DNN	BNN	BaLONN
Optimizer	Adam	Adam	Adam
Learning Rate	0.08	0.008	0.008
Loss function	MSE	negloglik	negloglik
Trainable Prior	-	True	True
Epochs	400	300	300

### 3.1 Evaluation metrics

The fact that we are dealing with uncertainties implies that the result is non-deterministic, i.e., the outputs can consist of a sample of several plausible outputs. For instance, for some patients, the evaluation of the expected therapy time can be a range (for instance, the expectation is that the patient will get a medium to long therapy time TL), and not an exact outcome (either a medium OR a long TL). In a real clinical environment, this ambivalent scenario is much more realistic than a pure deterministic one. For this reason, the validation must be performed using distributed outputs.

To compute the final validation and its corresponding statistics, we define the true prediction for each patient i ( ${\mathrm{Pr}}_{i}$ ) for both HF and TL as the statistical value that best matches the expected target value (with M the total number of patients). For the computation of ${\mathrm{Pr}}_{i}$ , we perform a micro statistic, where the average of the predicted values $f_{ij}$ is compared to the target value $O_{i}$ , where j is the number of times that the statistical model is run in order to obtain and sample the different predictions, and N is the total number of times the model is run to generate a sample of predictions (as has been shown in Figure 4). In our implemented models, N=1000 .

In our validation, we opt to use micro averages^14^, i.e., averages biased by class frequency. To this end, we must count the number of true predictions obtained after sampling the outcomes from the statistical model. If the distance between the average and the target lies below a tolerance value, then this state will be considered as a true prediction ${\mathrm{Pr}}_{i}^{m}=1$ , where m refers to the class we want to predict: if we compute HF, then m=1 , otherwise TL corresponds to m=2 .

$${\mathrm{Pr}}_{i}^{m}=\left\{\begin{array}{cc} 1, & if\phi_{i}^{m}=\frac{\sum_{j=1}^{N}f_{ij}}{2}-O_{i}<Tr\\ 0, & otherwise \end{array}\right.$$ (3)

Since we are simultaneously dealing with a binary (HF) and a multiclass (TL)^15^ prediction, we require an adaption of the prediction ${\mathrm{Pr}}_{i}^{m}$ implemented in the model validation:

• For ${Tr}_{HF},$ we consider the prediction of a binary value between 0 and 1, such that 0<ϕ<1.0 , such that ${Tr}_{HF}=0.7$ is a reasonable definition to sample a majority of one of the two binary values.
• For ${Tr}_{TL},$ we are evaluating the distance between different states between 0 and 3. Contrary to the binary prediction (which can be either 0 or 1) we can accept predictions with more than two values, for instance 2 and 3 (expectative of a medium to large therapy time), such that 0<ϕ<2 ; according to this, ${Tr}_{TL}=1.2$ is a reasonable definition to sample at least two plausible model outputs.

In a nutshell, Tr is a parameter to decide how many simultaneous states from the output $f_{ij}$ can be accepted and is the accepted degree of variability of the model. Based on these two definitions, we can then compute the precision as

$$P^{m}=\frac{\sum_{i=1}^{M}{\mathrm{Pr}}_{i}^{m}}{M}$$ (4)

while the total error was estimated as:

$$E=1-\frac{\overset{M}{\underset{i=1}{\sum }}{\phi_{i}}^{m}}{M}$$ (5)

### 3.2 Model performance

To test the correct functionality of the models, we perform a first inspection of the prediction’s distribution. For the baseline model (NNs), we employ the predicted values, while for the BNN, we compute the mean value from the sampled predictions. This first general result indicates, first, that the model has variability and, second, that it can reproduce the test data dynamics.

**FIGURE 5:**
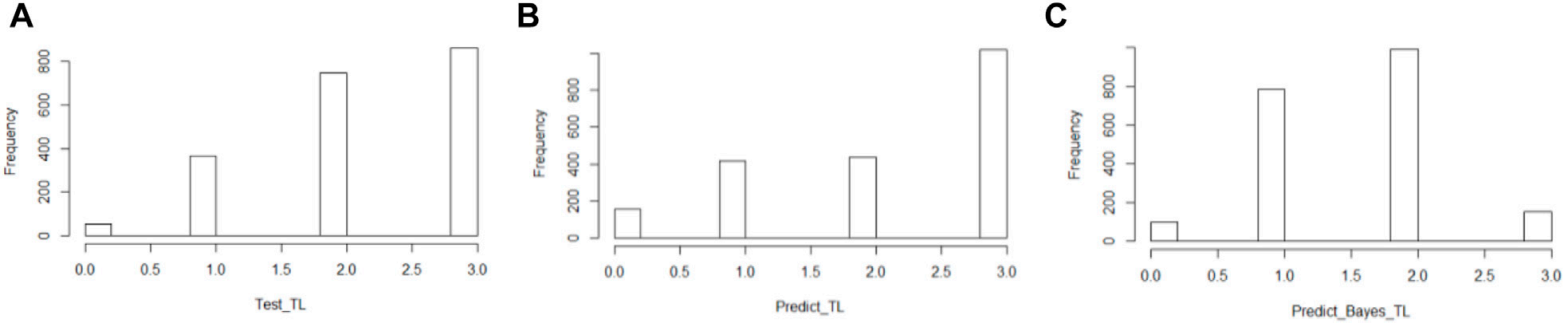
Distribution of the therapy length (TL) of the target data **(A)**, the prediction computed with the baseline model **(B)**, and the BNNs **(C)**.

Furthermore, we analyze the predicted variability and check if it is generated by both the model structure and the information integration, and not by the stochastics. As a result of a first inspection, we discovered that the mean value distribution of the sampled predictions derived by BNNs is different from the target distribution (Figure 5), which samples the absolute values. As a result, the way in which the uncertainty of the outputs is modeled can affect the overall distribution of the prediction.

Our next step was to analyze the model performance by sampling different predictions for each patient. Before applying this concept to BaLONNs, we explored the performance of BNNs to better understand their behavior. It is necessary to carry out a detailed validation of the predictions, considering the possibility of ambiguous results, as shown in Figure 3. It is possible to get a short or middle expected TL for a single patient, but the distribution gives more weight to one of the prognoses, as shown in Figure 5. Therefore, the main expectation is a short TL, with a low chance of a middle TL. A boxplot can be used to represent the predicted outcomes of a patient population, as opposed to just a single prediction.

**FIGURE 6:**
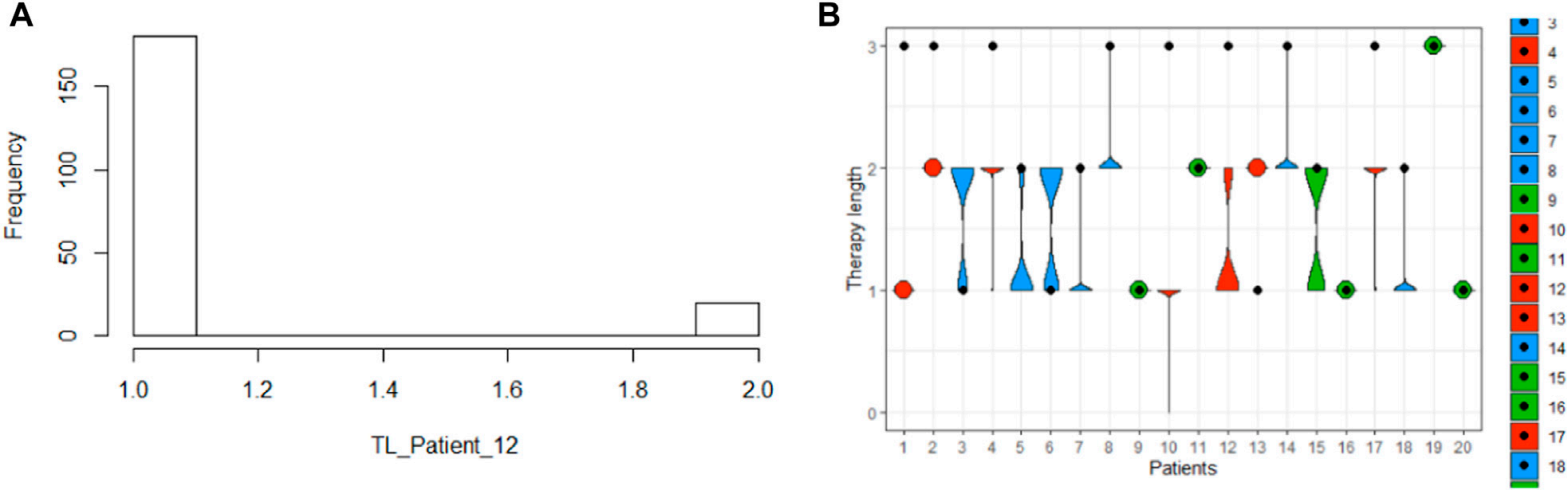
Precision of the prognosis of the therapy length for one patient with an ambiguous output **(A)**, and for a patient cohort (20 patients) in the whole population with validation results: green is a perfect prognosis, blue is a by-prognosis that is still fulfilled inside the uncertainty of the prediction, and red is an off-prognosis. The black points are the targets.

In Figure 6, we deploy this boxplot for a small fraction of the patient population and measure the distance to target values to validate the model. The color keys represent the quality of the validation. Green stands for a perfect validation, blue for a prediction where the target value lies inside the distribution of the predictions, and red for an incorrect prediction, where the target lies outside the distribution of the computed predictions.

**FIGURE 7:**
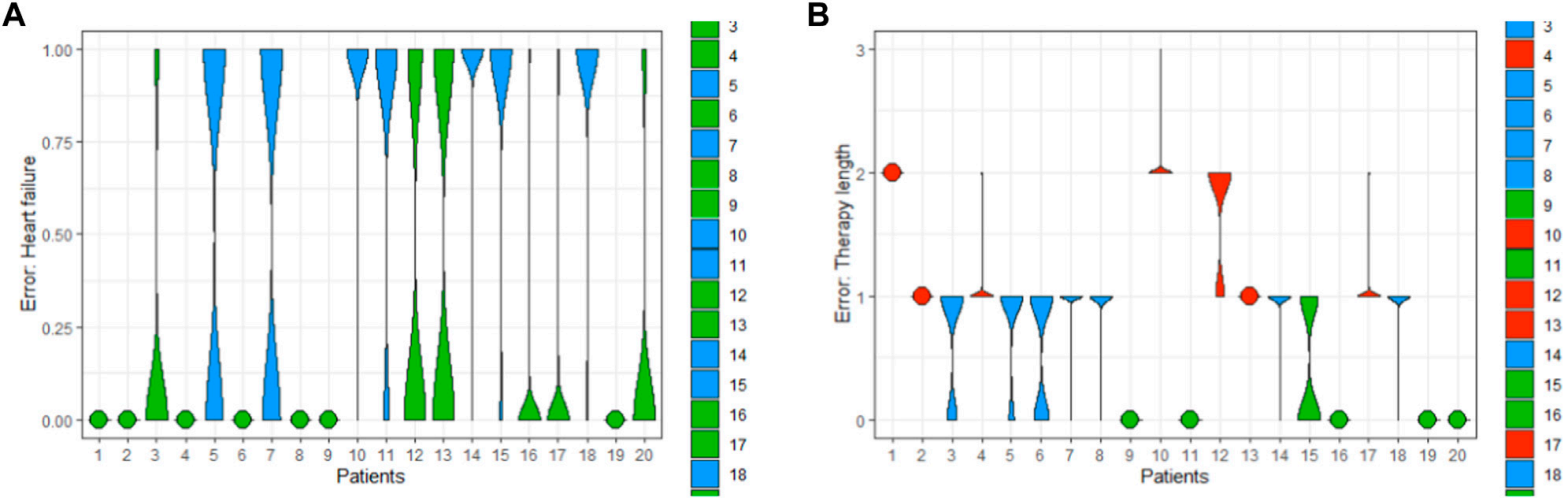
Exemplary error of the predictions of HF **(A)** and TL **(B)** computed for a patient cohort (20 patients).

The computation of the error considers the fact that both observables, therapy length and class of heart failure, are distributed as well. Finally, the results can be either represented using box plots or violin plots (Figure 7).

These kinds of visualizations are useful ways to qualitatively understand how the implemented models are working.

• Green when a model is relatively confident,
• Blue when the model delivers a prognosis with high uncertainty,
• And red, when the uncertainty is extremely large.

Of course, this has an implication for the final validation: with this implementation, we were able to reduce the error to *24.48*% and *21.24%*, representing an improvement of about *3%* with respect to pure deterministic models (total error of baseline model was 37.7%). But this error reduction has been obtained because we account predictions “in blue”, i.e., we are accepting off-predictions, where the targets lie inside the predicted distribution. Otherwise, the computed predictions of the BNNs only considering the true positives/negatives could deliver a much higher prediction error (see Figure 7).

### 3.3 Parameter analysis

**FIGURE 8:**
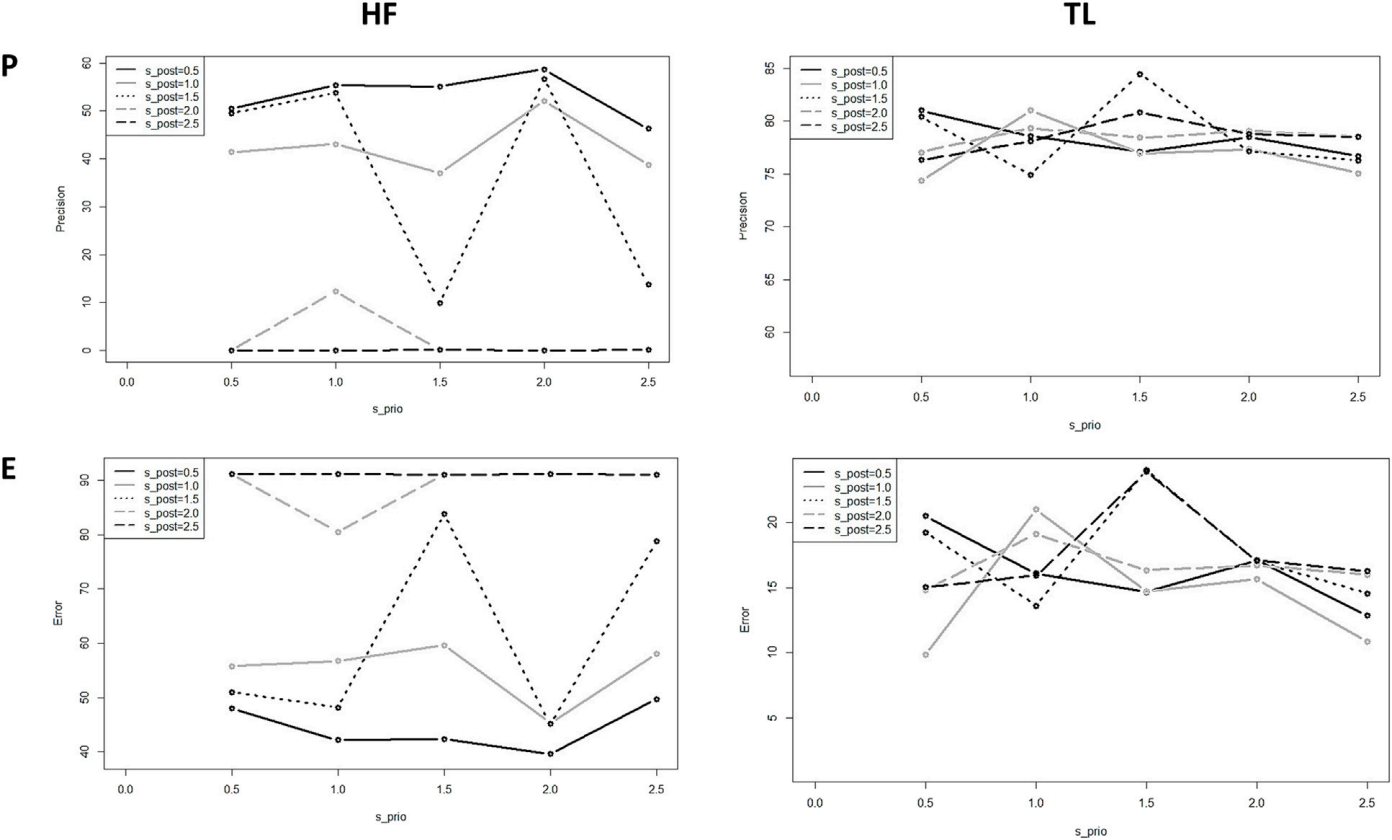
Precision and error of the predicted HF and TL using BNNs for different standard deviations; $\sigma_{\mathrm{pr}}$ (*x*-axis), and $\sigma_{po}$ (different shapes).

To get a better insight into the performance of the model, we assessed the model sensitivity to the standard deviation of the prior, $\sigma_{\mathrm{pr}}$ , and of the posterior, $\sigma_{po}$ and in this way, we analyzed the role of the distribution of the prior/posterior in the final result (Figure 8). This iteration is also a test of the robustness of the model by changing conditions or inputs, which is equivalent to performing adversarial tests on trained networks. The computation of these results was computationally intensive (about 2 h) on a normal desktop. If we are dealing with large datasets, any parameter optimization considering this variation will require a significant amount of computational power.

In this test, we observe for almost all the values a high model sensitivity on the prior and posterior standard variations. Also, the precision obtained for *HF* is smaller than the precision obtained for TL, due that *HF* is a binary output and has fewer degrees of freedom than the multilabel output TL; we can understand the behavior of this state as a physical binary state that gets polarized beyond a critical value in the fluctuations of the system, represented by $\sigma_{po}$ . Thus, we observe that around $\sigma_{po}=1.5$ (and $\sigma_{\mathrm{pr}}\epsilon \left[0.5,2.5\right]$ ) the *HF* states get unstable, and for $\sigma_{po}>1.5$ the overall precision by *HF* dramatically decreases^16^ (see Figure 8).

Since the standard deviation influences the distribution of the weights in the model, it is to assume that this definition must reflect the distribution of the uncertainty in the training dataset. Otherwise, the model follows its own dynamics, deteriorating its ability to fit experimental targets.

From these results, we conclude that one plausible combination is $\sigma_{\mathrm{pr}}=1.5$ and $\sigma_{po}=1.0$ .

**FIGURE 9:**
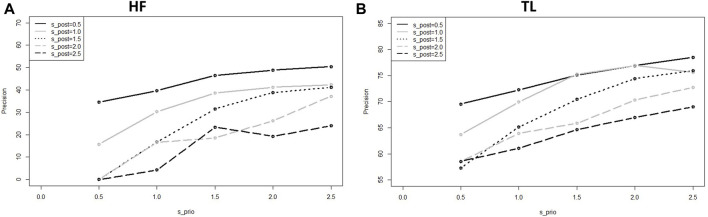
Computed precision of the BaLONNs for HF **(A)** and TL **(B)** and different standard deviations; $\sigma_{\mathrm{pr}}$ (*x*-axis), and $\sigma_{po}$ (different shapes).

When we tested the model sensitivity for the BaLONNs, we observed that there is a lower fluctuation of the precision/error values for $\sigma_{\mathrm{pr}}\epsilon \left[0.5,2.5\right]$ ) and $\sigma_{po}$ as the control parameter (Figure 9). Like a physical system, by freezing the layers we are reducing the degrees of freedom of the network. This reduces in this way the stochasticity, and in general the instability of the trained network. Also, in this case, we have observed that the *HF* precision is much lower than *TL*. Different from the BNNs, BaLONNs do not accept prior layers as trainable, which probably contributes to the deterioration of the HF validation.

We obtain acceptable validations for $\sigma_{\mathrm{pr}}=1.5$ and $\sigma_{po}=1.0$ . We finally computed the final predictions in a single computation. For this computation, we considered a *HF* tolerance of 0.6 (for the loop the *HF* tolerance was 0.6) and made an average of over 1,000 models (see Table 5).

**TABLE 5:** Validation results for the baseline model, BNNs and BaLONNs. For the stochastic models, we have averaged over 1,000 different models.

	NN-baseline model	BNN	BaLONN
E (%)	60.9	43.5	43.59
	4.73	15.13	12
Tot E (%)	32.81	29.31	27.79
Pr (%)	42.05	65.69	61.42
	55.08	80.15	77.14
Tot. Pr (%)	48.56	72.92	69.28

The modeling of epistemic uncertainty increases the precision of the modeling, simply because we are accepting ambiguous results as true predictions inside the tolerance range. In contrast, the base model is trying to make an exact and predictive prediction based on the training data. This effect reflects the fact that the inherent stochastics play a relevant role when a prediction is computed.

Furthermore, we observed that the validation of *HF* using BaLONNs does not get the same quality as a fully trainable model, despite the mean error lying below the mean error of BNNs. Since the logic gates are frozen/non-trainable parameters, the whole model has fewer degrees of freedom, limiting the possibility that the model converges to the target values. In this case, we face again the typical tradeoff problem: either to accept dealing with interpretable models with less accuracy or to have an accurate model with less interpretability.

We have also observed that while the precision of HF gets increased by adjusting the model’s hyperparameters, the TL’s precision decreases. The integration of these parameters in a single model is to some degree problematic, firstly considering that both parameters have a different number of states, and secondly, because their coupling is weak, i.e., TL depends on HF as well as additional parameters that are not integrated into the model. This was in particular evident in the NN-baseline model. Despite this, we could find a reasonable tradeoff in the parameter selection to deliver acceptable accuracy for both parameters.

## 4 Discussion

The definition of explainability usually focuses on methods to make the outcomes from black-box models, like DNNs, interpretable. Lime and SHAP, for example, use local explainability to build surrogate models for black box machine learning models to provide them with interpretability^17^. In our approach, we have opted for a different strategy by implementing nodes in the DNN model that can be defined as logical operators, LONN, which are interpretable. By doing this, we set up a hierarchy of parameters in the input layers that can then be logically combined, without requiring additional surrogate models.

In a nutshell, the concept of explainability in AI requires a particular taxonomy.

i. **Computational Explainability:** AI-explainability on a computational level refers to the correlation between model outputs and inputs, i.e., the evaluation of the plausibility of model predictions in the context of the inputs (Linardatos et al., 2020b).

Similarly, the Shapley index, a concept borrowed from the Game Theory, evaluates how individual input features have a marginal contribution (in game theory, how much individual features “cooperate” to get a final output^18^) on the final mode output, a concept frequently used in machine learning.

ii. **Instances of Explainability:** Additionally, Bayesian-NNs^19^ are applied to analyze instances of explainability, i.e., to account uncertainties in predictions.
iii. **Structural Explainability:** Finally, explainability can be also understood in terms of what currently the model is computing, i.e., by understating the internal processes in the model’s architecture, and how the model automatically generates a hierarchy of input parameters, a concept that has been already developed for deep neural networks (LoNNs—cite first article)

Clearly, our implementation belongs to the third class of explainability, where we are trying to gain more insight into how the computing process takes place.

However, the problem of *structural explainability* does not only revolve around how information is integrated and how the corresponding outputs are computed based on the interconnectedness of the inputs. It also concerns the implicit handling of uncertainty in the data. This factor is particularly relevant in the medical field, where biological variability has an influence on the development of a disease. Thus, while for one patient a specific diagnosis or outcome can clearly be established after therapy, for another patient there may be variability that cannot simply be ignored and that affects both the diagnosis and the assessment of the outcome of therapy. Therefore, interpretability must consider not only the information flow and output computation in a model but also the fact that in certain cases it is not possible to estimate an exact value. As a result, models taking epistemic uncertainty and explainability into consideration, such as our BaLONNs, aim to continuously inform the customer when the model cannot behave deterministically and when the prediction it makes is uncertain.

We believe that the inclusion of this kind of variability in assistant systems, like recommender or expert systems in medicine, is an important step, also in the design of human-centered solutions. Several studies have shown that trusting *digital systems beyond their capacity and functionality can present high actual costs, as well as more nuanced effects of compromising organizational integrity and personal security* (Hardré et al., 2016). This is, in particular, relevant in the medical field, where the pressure in daily business might lead to strongly relying on automatic systems (Goddard et al., 2012), which, despite being well-validated, could lead to wrong decisions when customers assume that the outputs are precise when in reality are not.

Our novel approach is essentially an integration of LONNs with probabilistic layers. Our preliminary results have demonstrated that this approach can fulfill its goal: we are able to get acceptable validation values as well as acceptable precision in the prediction of the two parameters used in this study, namely, the prediction of the kind of heart insufficiency (HF) and the expected therapy time of the patients (TL). However, the precision of the model lies below a conventional deep learning model (which can be much more precise if we increase the number of layers). As we have found in a previous study, explainability implies a tradeoff between model performance and precision: while explainable models are often small and constrained to few elements to keep them explainable, the expected precision is in this case significantly lower than for classical black-box models^20^. The customer must decide at the end how much precision she wants to sacrifice in exchange for a simple model.

The potential next step in this work is to introduce a level of stochasticity not only in the extreme layers but also in the logical switches, as well as in the number of these switches. In this way, we generate different explainable models, assuming that not only one but a family of explainable models is able to model the data. An additional analysis, not performed in this study, is the relation between stochasticity and minority oversampling using SMOTE, i.e., if by implementing SMOTE the underlying epistemic uncertainty of the training datasets gets distorted. Finally, the handling of stochasticity could be computationally intensive when large datasets are analyzed; perhaps alternative methods, like quantum computing, could be helpful to generate more efficient stochastic BaLONN models from large datasets.

## 5 Conclusion

In this work, we presented a novel modeling technique that combines a *structural explainable* model architectures based on logic neural networks (LONNs) and Bayesian methods in a single BaLONN model. In this way, we improve user-centered solutions that inform users when they are uncertain about a prediction, in this case, the prediction of therapy based on physiological parameters. Unlike other solutions, this approach does not nudge customers to accept the model’s predictions. While this solution does not present an answer to well-founded critics against AI as technology, for instance as a surveillance tool (Fulterer, 2022), it is at least a way to compensate for well-known deficits in the implementation of this technology, making it user-centered (in our case for physicians) by deploying results that can be distrusted by the user. By doing so, we can avoid blindly relying on models to deliver high-quality results^21^.

We demonstrated that our model reaches a precision of 69%, considering that some of the true predictions are ambiguous but lie in the tolerance range of acceptability. This precision lies below the 70% precision of Bayesian neural networks. However, we think that the slight reduction in precision is an acceptable tradeoff considering that the architecture of the NN is composed of a few neurons and that some of these neurons are non-trainable.

This result is thus a relevant basis for the development of assistant and expert methods, for instance, recommender systems, in critical fields like medicine where the customer’s work should not be fully automated, and his expertise is continuously required.

## Data Availability

The original contributions presented in the study are included in the article/supplementary materials, further inquiries can be directed to the corresponding author.

## Footnotes

1
https://healthinfoservice.com/most-common-icd-10-error-codes/

2
https://www.heise.de/hintergrund/Wie-eingebautes-Misstrauen-KI-Systeme-sicherer-machen-kann-6049154.html?wt_mc=rss.red.ho.ho.atom.beitrag.beitrag

3 The data has been obtained from the UCI repository: https://archive.ics.uci.edu/ml/datasets/Heart+failure+clinical+records

4
https://www.r-bloggers.com/generating-synthetic-data-sets-with-synthpop-in-r/

5
https://scholarworks.utep.edu/cgi/viewcontent.cgi?article=2170&context=cs_techrep

6 In case of evaluation of time series, for instance in ECGs, we expect to find aleatoric uncertainty

7
https://cedar.buffalo.edu/∼srihari/CSE574/Chap5/Chap5.7-BayesianNeuralNetworks.pdf

8
https://blogs.rstudio.com/ai/posts/2019-06-05-uncertainty-estimates-tfprobability/

9
https://en.wikipedia.org/wiki/Evidence_lower_bound

10
https://xyang35.github.io/2017/04/14/variational-lower-bound/

11
https://blogs.rstudio.com/ai/posts/2019-11-07-tfp-cran/

12
https://blogs.rstudio.com/ai/posts/2019-06-05-uncertainty-estimates-tfprobability/

13
https://www.mathworks.com/help/stats/prob.normaldistribution.negloglik.html

14
https://stats.stackexchange.com/questions/156923/should-i-make-decisions-based-on-micro-averaged-or-macro-averaged-evaluation-mea

15 In this work we are dealing with both a multiclass and a multilabel model: with the single model outputs, we are dealing with a multiclass classification, while after sampling all the outputs we are dealing with a multilabel classification.

16 This observation resembles a phase transition, where $\sigma_{po}$ has influence on the HF states. Up to a $\sigma_{po}>2.5$ all the states HF are frozen, i.e., the model has no variability, and the outputs are fixed to a single state.

17
https://towardsdatascience.com/idea-behind-lime-and-shap-b603d35d34eb

18
https://towardsdatascience.com/the-shapley-value-for-ml-models-f1100bff78d1

19
https://towardsdatascience.com/bayesian-neural-network-7041dd09f2cc

20
https://www.interpretable.ai/interpretability/what/

21 This corresponds to a kind of experimenter bias, where model expectations supported by validated models can distort the real interpretation of the model’s output.

## Abbreviations

EHR; Electronic Health Record, NN; Neural Network, DNN; Deep Neural Network, BNN; Bayes Neural Network, LONN; Logic Neural network, BaLONN; Bayesian logical Neural Network, BNN; Bayesian Neural Network, HF; Heart Failure, TL; Therapy Length.
